# Interleaved Pro/Anti-saccade Behavior Across the Lifespan

**DOI:** 10.3389/fnagi.2022.842549

**Published:** 2022-05-18

**Authors:** Rachel Yep, Matthew L. Smorenburg, Heidi C. Riek, Olivia G. Calancie, Ryan H. Kirkpatrick, Julia E. Perkins, Jeff Huang, Brian C. Coe, Donald C. Brien, Douglas P. Munoz

**Affiliations:** ^1^Centre for Neuroscience Studies, Queen’s University, Kingston, ON, Canada; ^2^Department of Medicine, Queen’s University, Kingston, ON, Canada; ^3^Department of Biomedical and Molecular Sciences, Queen’s University, Kingston, ON, Canada

**Keywords:** inhibitory control, anti-saccade, interleaved, lifespan, change point analysis

## Abstract

The capacity for inhibitory control is an important cognitive process that undergoes dynamic changes over the course of the lifespan. Robust characterization of this trajectory, considering age continuously and using flexible modeling techniques, is critical to advance our understanding of the neural mechanisms that differ in healthy aging and neurological disease. The interleaved pro/anti-saccade task (IPAST), in which pro- and anti-saccade trials are randomly interleaved within a block, provides a simple and sensitive means of assessing the neural circuitry underlying inhibitory control. We utilized IPAST data collected from a large cross-sectional cohort of normative participants (*n* = 604, 5–93 years of age), standardized pre-processing protocols, generalized additive modeling, and change point analysis to investigate the effect of age on saccade behavior and identify significant periods of change throughout the lifespan. Maturation of IPAST measures occurred throughout adolescence, while subsequent decline began as early as the mid-20s and continued into old age. Considering pro-saccade correct responses and anti-saccade direction errors made at express (short) and regular (long) latencies was crucial in differentiating developmental and aging processes. We additionally characterized the effect of age on voluntary override time, a novel measure describing the time at which voluntary processes begin to overcome automated processes on anti-saccade trials. Drawing on converging animal neurophysiology, human neuroimaging, and computational modeling literature, we propose potential frontal-parietal and frontal-striatal mechanisms that may mediate the behavioral changes revealed in our analysis. We liken the models presented here to “cognitive growth curves” which have important implications for improved detection of neurological disease states that emerge during vulnerable windows of developing and aging.

## Introduction

Inhibitory control, or the ability to voluntarily suppress prepotent responses in favor of more appropriate and adaptive ones, is a critical executive function that enables goal-driven behavior in everyday life (Miyake and Friedman, 2012; Diamond, 2013). The capacity for inhibitory control can be measured with numerous behavioral paradigms and has been shown to change dynamically over the course of the lifespan; it is deficient in early childhood, improves dramatically throughout adolescence, remains relatively stable from young to mid adulthood, then declines gradually later in life (Munoz et al., 1998; Bedard et al., 2002; Klein et al., 2005; Sebastian et al., 2013a; Ferguson et al., 2021). This cognitive trajectory, which typically follows a curvilinear U-shape, is paralleled by changes in the structure and function of brain regions that mediate inhibitory control, namely, those involved in frontal-parietal and frontal-striatal circuits (Aron, 2011; Swick et al., 2011; Sebastian et al., 2013b). Importantly, abnormalities in these circuits occurring during vulnerable windows of brain maturation or decline can lead to the onset of neurological disorders of inhibitory control (i.e., attention-deficit hyperactivity disorder, Parkinson’s disease) at either end of the lifespan (Durston et al., 2011; Pagonabarraga and Kulisevsky, 2012). Improved identification and understanding of these vulnerable windows requires robust characterization of inhibitory control across normative development and aging. Here we describe the use of a simple and sensitive eye tracking paradigm of inhibitory control (the interleaved pro/anti-saccade task) to investigate changes in this important cognitive process from early childhood through to old age. We first introduce the basic parameters of this task, the behavior it produces, and the neural mechanisms underlying those behaviors. We then outline how existing work using this task to study development and aging can be expanded upon by considering age continuously across the lifespan and employing flexible modeling techniques.

As compared to traditional manual-response tasks, eye tracking paradigms provide a more direct means of assessing the neural circuitry underlying inhibitory control that are also easily understood by young children and older adults. In the anti-saccade (ANTI) task (Hallett, 1978), participants are required to suppress the reflexive response to look at a peripherally appearing visual stimulus and look in the opposite direction instead. This is in contrast to the pro-saccade (PRO) task, where conditions are nearly identical but participants are required to look at the stimulus as soon as it appears. As the location of the stimulus and the saccade goal are decoupled in the ANTI task, successful execution requires top-down inhibition of the reflexive response to look at the stimulus, followed by a transformation of the stimulus location into a voluntary motor command to look in the opposite direction (Munoz and Everling, 2004). These additional steps necessitate higher-order cognitive control and lead to longer saccadic reaction times (SRT; time between stimulus appearance and saccade onset) in ANTI as compared to PRO tasks (Munoz and Everling, 2004; Coe and Munoz, 2017). If top-down inhibition is insufficient in the ANTI task, a direction error (an erroneous PRO toward the stimulus) will be triggered.

Experimental manipulations of the PRO and ANTI tasks produce distinct changes in SRT and direction error rate. In the gap condition, removal of the central fixation point 200 ms before stimulus appearance elicits increased rates of “express saccades,” reflexive, short-latency saccades that approach the minimum sensory-motor conduction delays in the brain (Fischer and Boch, 1983; Fischer and Ramsperger, 1984; Paré and Munoz, 1996). Other manipulations, such as increasing the temporal and spatial predictability of stimulus appearance, have also been shown to increase the frequency of these short-latency saccades (Dorris and Munoz, 1998; Bibi and Edelman, 2009; Marino and Munoz, 2009). In the interleaved PRO/ANTI task (IPAST), PRO and ANTI trials are randomly interleaved within a block, with trial condition indicated by the color of the central fixation point. The IPAST requires continuous updating of the saccade goal from trial to trial, producing longer SRTs and increased direction error rates, relative to blocked tasks (Cherkasova et al., 2002; Reuter et al., 2006). The IPAST with gap is therefore highly effective at eliciting correct responses and direction errors made at both express and longer (often referred to as “regular”) latencies. The SRT distribution for PRO trials in this task consists of both express- and regular-latency correct responses, while the SRT distribution for ANTI trials in this task consists of express- and regular-latency direction errors, as well as regular-latency correct responses (Munoz and Everling, 2004; Coe and Munoz, 2017).

The neural circuitry underlying these saccade behaviors is well-characterized, and includes areas of the frontal and parietal cortices, basal ganglia (BG), thalamus, superior colliculus (SC), brainstem, and cerebellum (Hikosaka et al., 2000; Scudder et al., 2002; Sparks, 2002; Munoz and Everling, 2004; Schall, 2004; McDowell et al., 2008; Watanabe and Munoz, 2011). Briefly, the appearance of the peripheral stimulus induces a transient visual response that enters the brain via retino-geniculo-striate and retino-tectal pathways. This visual response propagates through several frontal-parietal and frontal-striatal circuit structures, including the frontal (FEF), supplementary (SEF) and parietal (PEF) eye fields, dorsolateral prefrontal cortex (DLPFC), and BG, before converging on the SC. From the SC, the signal to either initiate or suppress a saccade is projected directly to the brainstem reticular formation. We have previously proposed that on PRO trials, the transient visual response either drives an express-latency saccade via a direct sensory-motor transformation, or a regular-latency saccade via propagation of a well-learned, automated motor command (Munoz and Everling, 2004; Coe and Munoz, 2017). On ANTI trials, two different types of suppression are required to prevent the express- and regular-latency direction errors from being triggered. Prior to stimulus appearance, pre-emptive, global inhibition is required to suppress the direct sensory-motor transformation of the visual transient. If this first suppression fails, an express-latency error is triggered. After stimulus appearance, the voluntary, location-specific motor command to make an ANTI must override the automated motor command to make a PRO. If this second suppression fails, a regular-latency error is triggered (Coe and Munoz, 2017; Coe et al., 2019).

Existing work using PRO and ANTI tasks to study development and aging highlights the sensitivity of these tasks to changes in underlying frontal-parietal and frontal-striatal circuitry as a function of age. Children as young as 5 years old can perform these tasks, but have long and variable SRTs and high direction error rates (Munoz et al., 1998; Fukushima et al., 2000; Klein and Foerster, 2001; Kramer et al., 2005). Task performance improves throughout childhood and adolescence, with peak, adult-level behavior suggested to emerge from the ages of 12–15 (Fukushima et al., 2000; Klein and Foerster, 2001; Luna et al., 2004; Irving et al., 2006; Bucci and Seassau, 2012), and consistently short SRTs and low direction error rates being maintained from the ages of 18–25 (Munoz et al., 1998; Fukushima et al., 2000; Klein and Foerster, 2001; Luna et al., 2004; Velanova et al., 2008; Alahyane et al., 2014). Performance appears to decline more gradually from young to mid adulthood (Munoz et al., 1998; Irving et al., 2006), while in the seventh decade of life onward, increases in SRT and direction error rate become more pronounced (Munoz et al., 1998; Klein et al., 2000; Sweeney et al., 2001; Abel and Douglas, 2007; Fujiwara et al., 2010; Peltsch et al., 2011; Noiret et al., 2016; Fernandez-Ruiz et al., 2018).

The contribution of this literature notwithstanding, previous studies are limited in that they investigate developing and aging cohorts separately, compare individuals grouped into small, artificially delineated age bins, and utilize different task parameters (i.e., gap vs. no-gap, interleaved vs. blocked design) and pre-processing methods. Using age as a continuous predictor variable in regression models allows for a more precise characterization of age-related effects on saccade behavior. This has been done in a number of developing (Luna et al., 2004; Ordaz et al., 2010; Bucci and Seassau, 2012; Alahyane et al., 2014), aging (Mack et al., 2020; Coors et al., 2021), and lifespan (Klein et al., 2005) cohorts to-date. The conventional linear regression models used in many of these studies, however, may be insufficiently flexible to capture the complex, non-linear trajectories of age-related changes in the brain (Fjell et al., 2010). Semiparametric regression models, such as those that rely on smoothing splines, have been demonstrated to be more robust in this regard (Fjell et al., 2010, 2013; Nook et al., 2020; Sørensen et al., 2021), and have recently been used to identify the ages at which various behavioral and brain-based measures undergo significant periods of change (Simmonds et al., 2014; Wierenga et al., 2019; Calabro et al., 2020; Nook et al., 2020; Calancie et al., 2021).

The goal of the present study is to investigate the effect of age on IPAST behavior and identify significant periods of change throughout development and aging. We use IPAST data collected from a large cross-sectional cohort of normative individuals, standardized pre-processing protocols, generalized additive models, and change point analysis to robustly characterize changes in inhibitory control across the lifespan. We hypothesize that IPAST behavior will follow a curvilinear U-shaped trajectory of improvement, maturation, and decline, and that considering PRO and ANTI behaviors made at express- and regular-latencies, as well as voluntary override time–a novel measure describing the time at which voluntary processes overcome automated processes on ANTI trials–will further differentiate developmental and aging processes. We consider the identified behavioral changes in relation to converging animal neurophysiology, human neuroimaging, and computational modeling literature which provide insight into the neural mechanisms underlying inhibitory control across the lifespan.

## Materials and Methods

### Participants

All experimental procedures were reviewed and approved by the Queen’s University Health Sciences and Affiliated Teaching Hospitals Research Ethics Board. Healthy individuals between the ages of 5–93 were recruited from the greater Kingston area via newspaper and online advertisements. All participants reported no history of neurological or psychiatric illness and had normal or corrected-to-normal vision. A subset of participants aged 18 and older completed a Montreal Cognitive Assessment (MoCA), a brief screening tool shown to be sensitive in the detection of mild cognitive impairment (Nasreddine et al., 2005). Here, participants were excluded if they scored <20 on the MoCA. This cut-off score was determined based on the range of MoCA scores from the available subset of adult participants (aged 18–93) in our study cohort prior to outlier rejection (see Section “Pre-processing”). The use of a cut-off score lower than the recommended 26 (Nasreddine et al., 2005) is consistent with more recent studies suggesting that lower thresholds may decrease the false positive rate for mild cognitive impairment in large, diverse cohorts including older adults and individuals with lower education levels (Rossetti et al., 2011; Carson et al., 2018). Written informed consent was obtained from all individuals aged 18 and older. Written informed assent, in addition to a parent or guardian’s written informed consent, was obtained from all individuals under the age of 18. Study sessions took approximately 1 h each. Participants were compensated $20 CAD for their time.

### Recording and Apparatus

During the eye tracking portion of the study, participants were seated in a dark room with their heads resting comfortably in a head rest. Participants were seated 60 cm away from a 17 inch 1280×1024 pixel resolution LCD computer monitor. An infrared video-based eye tracker (Eyelink 1000 Plus, SR Research Ltd., ON, Canada) was used to track monocular eye position at a sampling rate of 500 Hz. A 9-point array calibration and validation procedure was performed for each participant prior to beginning the task to map raw pupil position into gaze position. Eyelink 1000 measures validation accuracy as the average error in degrees between gaze and validation target positions. Here, participants had to have an average validation accuracy < 1.5° in order for their eye tracking data to be considered sufficiently accurate for further analysis.

### Experimental Paradigm

**FIGURE 1:**
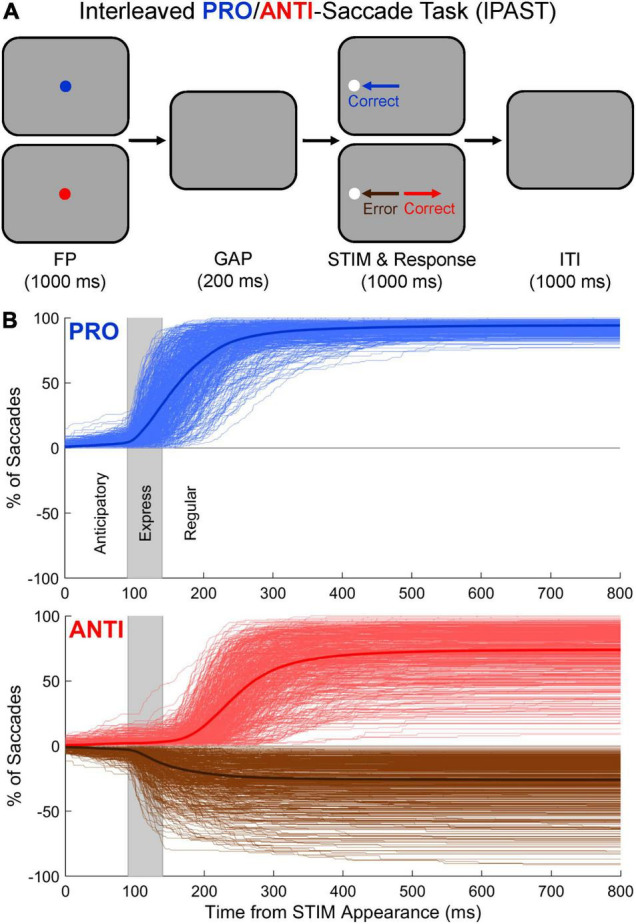
**(A)** Visual representation of the Interleaved PRO/ANTI-Saccade Task (IPAST). Each trial began with the appearance of a fixation point (FP) in the center of a black screen for 1000 ms. The color of the FP indicated the trial condition (green = PRO, red = ANTI). Following a 200 ms gap (GAP) during which the FP was removed, a gray stimulus (STIM) appeared 10° to the left or right of the FP position and remained on screen for an additional 1000 ms. On PRO trials, participants were instructed to look at the STIM as soon as it appeared. On ANTI trials, participants were instructed to look away from the STIM as soon as it appeared. Direction errors were saccades made toward the STIM on ANTI trials. An inter-trial interval (ITI) was presented for 1000 ms before the start of each new trial. Note that for illustration purposes, the colors of the FP, screen, and STIM shown in panel **(A)** differ slightly from how the task would appear to participants in the lab. **(B)** Cumulative SRT distributions for PRO and ANTI trials. On PRO and ANTI trials, saccades were classified based on when they occurred and their start and end positions. Saccades made toward the two potential STIM locations occurring between –110 and 89 ms relative to STIM appearance were considered “anticipatory” and excluded from further analysis. Saccades made toward the two potential STIM locations occurring between 90 and 800 ms relative to STIM appearance were considered “viable” and further delineated based on their latencies. PRO viable correct responses and ANTI viable direction errors were divided into express (90–139 ms) and regular (140–800 ms) latencies. Thick lines are averaged distributions for the entire study cohort. Thin lines are individual participants. Vertical gray windows indicate the express-latency epoch.

The IPAST (Figure 1A) consisted of two blocks of 120 trials each, lasting approximately 20 min in total. Each trial began with the appearance of a colored fixation point (FP; 0.5° in diameter, 44 cd/m^2^) in the center of a black screen (0.1 cd/m^2^) for 1000 ms. The color of the FP indicated the trial condition (green = PRO, red = ANTI). Following a 200 ms gap during which the FP was removed (GAP), a gray stimulus (STIM; 0.5° in diameter, 62 cd/m^2^) appeared 10° to the left or right of the FP position and remained on screen for an additional 1000 ms. On PRO trials, participants were instructed to look at the STIM as soon as it appeared. On ANTI trials, participants were instructed to look away from the STIM (i.e., to its diametrically opposite position) as soon as it appeared. An inter-trial interval (ITI) consisting of a black screen (0.1 cd/m^2^) was presented for 1000 ms before the start of each new trial. Drift checks occurred every 40 trials to confirm the accuracy of eye tracking or to allow for re-calibration, if necessary. Trial condition (PRO/ANTI) and STIM location (left/right) were pseudo-randomly interleaved with equal frequency throughout each block. Verbal task instructions and 10–20 practice trials were provided to each participant prior to beginning the task in order to ensure comprehension.

### Data Analysis

#### Pre-processing

The standardized pipeline used to convert, clean, and pre-process the IPAST data collected for each participant has been described in detail elsewhere (Coe et al., 2022). Briefly, custom automated scripts written in MATLAB (The MathWorks Inc., Natick, MA, United States) were used to detect saccades on a trial-by-trial basis based on criteria for eye movement speed and duration. A dynamic speed threshold was defined for each trial as the mean plus 2.5 times the standard deviation of the background noise during fixation, with a minimum possible value of 20°/s. Eye movement speed had to remain above this threshold for 10 ms in order for saccade detection to occur. Detected saccades were then classified based on when they occurred, relative to STIM appearance, and their start and end positions (Figure 1B and Supplementary Figure 1B). Saccades made toward the two potential STIM locations occurring between −110 and 89 ms relative to STIM appearance (i.e., after FP offset but prior to visual processing of the STIM) were equally likely to be correct responses or direction errors, indicative of guessing behavior (Munoz et al., 1998). These saccades were considered “anticipatory” and excluded from further analysis. Saccades made toward the two potential STIM locations occurring between 90 and 800 ms relative to STIM appearance (i.e., after FP offset and visual processing of the STIM) were considered “viable” and further delineated based on their latencies (see Section “Measures of Interest”).

Following basic pre-processing, participant outlier rejection was performed using a three-step procedure. The first two of these steps are based on criteria for each participant’s trial counts. The IPAST consisted of 120 PRO and 120 ANTI trials. *Behavioral counts* for PRO and ANTI trials were defined as all trials for which eye tracking was not lost. Eye tracking loss was most commonly due to poor calibration/validation, excessive head movement, or excessive eye blinks. Behavioral counts were then divided into *non-compliance counts*, defined as all trials in which the participant never fixated the FP, made a random saccade, or made no saccade at all (i.e., were non-compliant to the task instructions), and *viable counts*, defined as all trials in which the participant made a correct response or direction error during the viable window (i.e., 90–800 ms). Behavioral counts therefore reflect all trials in which any measurable behavior was performed, while viable counts reflect all trials in which a task-relevant behavior was performed. In the first step of the outlier rejection procedure, participants were removed if they had a PRO or ANTI viable trial count < 30. This criterion was used to exclude participants who had insufficient data (either due to poor eye tracking, or inability or unwillingness to participate) to adequately characterize task performance. Second, participants were removed if they had a PRO or ANTI eye loss or non-compliance trial count > 20% of the total expected trial count. This criterion was used to exclude participants with task behavior atypical from that of a normative population (e.g., an individual with > 24 PRO or ANTI trials in which eye tracking was not lost, but no task-relevant behavior was performed). Third, participants were removed if they completed a MoCA (subset of individuals aged 18–93) and scored < 20, as previously described.

#### Measures of Interest

A number of IPAST measures were investigated in order to assess changes in inhibitory control across the lifespan. The cumulative (Figure 1B) and instantaneous (Supplementary Figure 1B) SRT distributions for PRO and ANTI trials illustrate the timing and frequency of some of these measures. PRO and ANTI viable correct SRT were calculated for each participant as the mean time between STIM appearance and the onset of a correct saccade occurring within the viable window. The delineation of this viable window into express- and regular-latency epochs has been described previously (Fischer and Boch, 1983; Fischer and Ramsperger, 1984). Although the timing of the express-latency epoch can be influenced by various task parameters (Dorris and Munoz, 1998; Bibi and Edelman, 2009; Marino and Munoz, 2009), and is therefore somewhat arbitrary, healthy human participants typically make reflexive, short-latency saccades within the range of 90–140 ms (Munoz et al., 1998, 2003). On PRO trials, viable correct responses were therefore further delineated into express-latencies, occurring between 90 and 139 ms, and regular-latencies, occurring between 140 and 800 ms. On ANTI trials, viable direction errors were similarly divided into express- (90–139 ms) and regular- (140–800 ms) latencies. Ratios of PRO express-latency correct responses, PRO regular-latency correct responses, ANTI express-latency direction errors, and ANTI regular-latency direction errors were calculated for each participant using their viable trial counts as denominators.

As described in Coe et al. (2019), by subtracting the cumulative SRT distribution of ANTI direction errors from that of ANTI correct responses (Figure 1B, brown and red curves), we can estimate the time at which voluntary processes begin to overcome automatic processes on ANTI trials, or the voluntary override time (VOT). A 7-point box shaped kernel was used to smooth this distribution. VOT for each participant was determined as the minimum point along this smoothed distribution occurring within the window of 90–400 ms relative to STIM appearance (Supplementary Figure 2). Our seven IPAST measures of interest therefore consisted of: (1) PRO viable correct SRT, (2) ANTI viable correct SRT, (3) PRO express-latency correct response ratio, (4) PRO regular-latency correct response ratio, (5) ANTI express-latency direction error ratio, (6) ANTI regular-latency direction error ratio, and (7) VOT.

#### Generalized Additive Models and Change Point Analysis

In order to assess the effect of age on the IPAST measures described above, generalized additive models (GAMs; Hastie and Tibshirani, 1986) were performed using the *mgcv* package in R (Wood, 2017). GAMs are generalized linear models in which the linear predictor consists of a weighted sum of *K* basis functions, which are typically cubic or thin-plate regression splines (Wood, 2003, 2017). GAMs hold a number of advantages over more conventional regression models that make them ideal for investigating the complex trajectories of age-related changes in the brain (Sørensen et al., 2021). As GAMs are semiparametric, they enable flexible, data-driven estimation of non-linear trends across time series data that are less susceptible to variations in the range and sampling of data points (Fjell et al., 2010; Simpson, 2018). To prevent overfitting, GAMs are regularized by a smoothing parameter, λ, which can be selected using a variety of automated methods (Wood, 2017; Simpson, 2018). These features are particularly important for the characterization of lifespan cohorts in which the shape of developmental and aging trajectories may not be known *a priori*. When compared to linear, quadratic, and cubic regression models, semiparametric regression models such as GAMs have been shown to provide a superior fit to various behavioral and brain-based measures sampled across the lifespan (Fjell et al., 2010, 2013; Nook et al., 2020; Sørensen et al., 2021).

Here, GAMs defined by a smoothed fixed effect of age were performed for each of the seven IPAST measures of interest. In order to meet the assumption of normality for use of a Gaussian conditional distribution, IPAST ratio variables (which were naturally zero or one inflated) were first transformed with a logit transformation (Warton and Hui, 2011) before being entered into GAMs. Restricted marginal likelihood maximization (REML) was used to estimate the smoothing parameter, λ, for each GAM, as it has been suggested to be the optimal approach (Wood, 2011). As described by Wood (2017), and expanded upon by Simpson (2018), statistically significant periods of change can be determined from GAMs through estimation of the first derivative and simultaneous confidence intervals of the fitted trend using posterior simulation. In this manner, significant periods of change are identified at the time points where the simultaneous confidence intervals of the first derivative do not contain zero (*p* < 0.05). This approach has been adopted in a number of recent studies to identify the ages at which behavioral and brain-based measures undergo significant periods of change (Simmonds et al., 2014; Wierenga et al., 2019; Calabro et al., 2020; Nook et al., 2020; Calancie et al., 2021). Here, we follow recent work from Calabro et al. (2020) in which posterior simulation was used to generate 10,000 GAM fits and their derivatives at 0.1-year age intervals. 95% confidence intervals were then generated from these simulated derivatives. These analyses were conducted using the *LNCDR* package in R (Tervo-Clemmens and Foran, 2022). We sought to determine if, and when, significant periods of age-related change occur throughout the lifespan for each of our seven IPAST measures of interest. Finally, Spearman’s correlations were conducted to investigate the pairwise relationships between each of our measures of interest, given their non-normal distributions. Standardized residuals derived from each measure’s GAMs were used to control for age.

## Results

### Study Cohort

**FIGURE 2:**
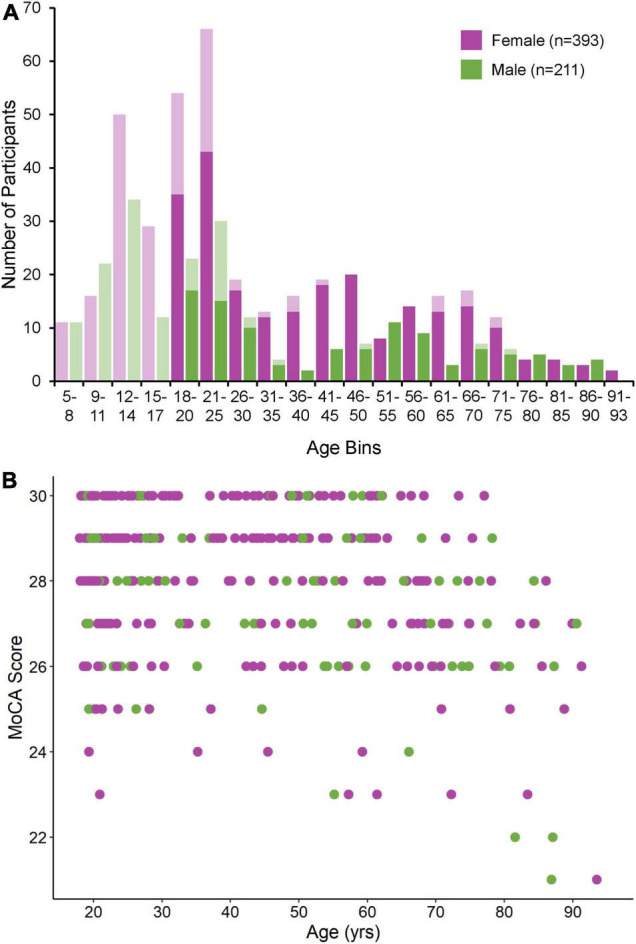
**(A)** Age distribution for male and female participants included in the final study cohort. Age bins vary in size from 3 to 5 years, with smaller bins used before the age of 20 and larger bins used afterward. Opaque bars indicate participants aged 18 and older for which MoCA scores were available. **(B)** MoCA score distribution for male and female participants depicted by the opaque bars in panel **(A)**.

631 individuals (409 F, 222 M, 5–93 years of age) were recruited to participate in this study from 2015 to 2022. Of the 430 individuals aged 18 and older, 346 (80%) completed a MoCA. We note that our original study cohort is skewed toward young (i.e., >25 years old) female participants due to extensive participant recruitment from local university and college student bodies, as well as our lab’s efforts to match control participants to various neuropsychiatric patient cohorts within this demographic. From this original study cohort, 26 participants were excluded as a result of our three-step outlier rejection procedure. Nine participants were excluded on the basis of a PRO or ANTI viable trial count < 30, 14 participants were excluded on the basis of a PRO or ANTI eye loss or non-compliance trial count > 20%, and three participants were excluded on the basis of a MoCA score < 20. One additional participant was excluded on the basis of not making a single ANTI viable correct response. The final study cohort therefore consisted of 604 participants (393 F, 211 M, 5–93 years of age). Of the 419 individuals aged 18 and older, MoCA scores were available for 335 (80%). The majority of individuals for which MoCAs were not available were between the ages of 18–25, a demographic for which scores in large population cohorts have been found to be well above the cut-off used here (Rossetti et al., 2011). Age and MoCA score distributions for male and female participants included in the final study cohort are shown in Figures 2A,B, respectively. Additional demographic information (i.e., education level, average MoCA score, where applicable) for all included and excluded participants is provided in Supplementary Table 1.

### Effect of Age on Interleaved Pro/Anti-saccade Task Behavior

Table 1 displays the GAM fit parameters (*ref df*, *F*, *p*, *R*^2^, deviance explained) for each of the seven IPAST measures of interest (see Section “Materials and Methods”). All measures exhibited significant (*p* < 0.05) age-related changes. The amount of deviance of the IPAST measures explained by age ranged from 7.18 to 31.2%. In order to investigate if GAM fits differed as a function of participant sex, a second GAM (Model 2) was defined by a smoothed fixed effect of age split by sex and also performed for each measure of interest. Bayesian information criterion (BIC) was used to compare the goodness-of-fit of Model 2 with the originally specified GAM (Model 1; smoothed fixed effect of age only), with lower values indicating a superior fit. Supplementary Table 2 displays the fit parameters for both models. For Model 2, each smooth fit of age remained significant when split by participant sex, and a similar amount of deviance of the measures was explained by age (7.31–31.5%). However, BIC values were lower (indicating a superior fit) for Model 1 for all measures investigated. We therefore describe the characteristics of these GAMs for the remainder of our results.

**TABLE 1:** GAM fit parameters.

IPAST measure	*Ref df*	*F*	*p*	*R* ^2^	Deviance explained
PRO Viable correct SRT	6.925	10.74	<2e-16	0.11	11.8%
ANTI Viable correct SRT	8.412	25.22	<2e-16	0.26	26.9%
PRO Express-latency correct response ratio	1.998	26.04	<2e-16	0.079	8.15%
PRO Regular-latency correct response ratio	1.005	46.33	<2e-16	0.0703	7.18%
ANTI Express-latency direction error ratio	6.106	13.9	<2e-16	0.124	13.1%
ANTI Regular-latency direction error ratio	7.857	31.88	<2e-16	0.293	30.1%
Voluntary override time	8.429	31.23	<2e-16	0.303	31.2%

### Significant Periods of Change Throughout Development and Aging

Significant periods of change for the fitted GAMs were identified at the ages where the confidence intervals of the first derivative did not contain zero (*p* < 0.05). At least one significant period of change was identified for each measure. With the exception of PRO express- and regular-latency correct response ratios, which exhibited significant change across the entire lifespan, all periods of change beginning before the age of 23 captured improvements in task performance (i.e., decreases in SRT/VOT or direction errors), while all periods of change beginning after the age of 23 captured declines in task performance (i.e., increases in SRT/VOT or direction errors). We therefore refer to periods of change as being either “developmental-related” or “aging-related,” depending on whether the period began before or after the age of 23.

#### Pro-saccade and Anti-saccade Viable Correct Saccadic Reaction Time

**FIGURE 3:**
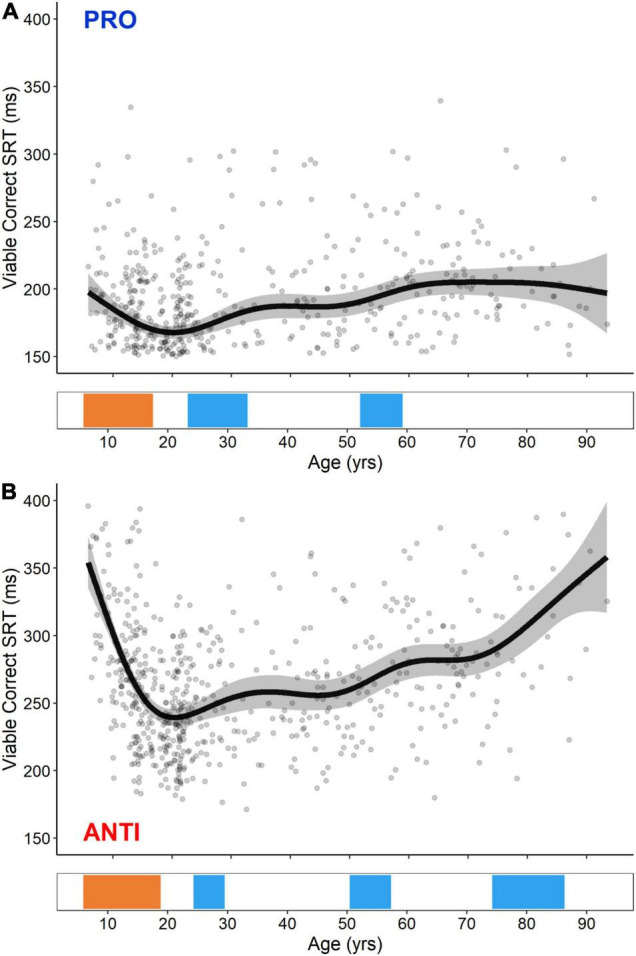
GAM fits and significant periods of change for PRO **(A)** and ANTI **(B)** viable correct SRT. Scatter points are individual participants, black curves are the GAM fits, and gray ribbons are the 95% confidence intervals. Bottom tiles indicate significant periods of developmental-related (orange) and aging-related (blue) change.

GAM fits and significant periods of change for PRO and ANTI viable correct SRT are shown in Figure 3. Previous studies that have used age as a continuous predictor in regression models of PRO and ANTI behavior have suggested that developmental trajectories are best characterized by an inverse curve fit, while aging trajectories are best characterized by a linear fit (Luna et al., 2004; Klein et al., 2005; Ordaz et al., 2010, 2013; Alahyane et al., 2014; Mack et al., 2020). The GAM fits presented here broadly support these claims, capturing a U-shaped trajectory of dramatic improvement (i.e., decreases in SRT) in childhood and adolescence, followed by a more gradual decline (i.e., increases in SRT) beginning in the third decade of life, which was steeper for ANTI SRT compared to PRO. Notably, however, the use of GAMs rather than more conventional approaches allowed us to capture these complex age-related processes within continuous, flexible models. Regarding change point analysis, both PRO and ANTI viable correct SRT exhibited significant developmental-related periods of improvement (i.e., decreases in SRT) beginning at the age of 5.8. For PRO SRT, this improvement continued until the age of 17.6, while for ANTI SRT, this improvement continued until the age of 18.7. Following these improvements, both measures exhibited multiple aging-related periods of decline (i.e., increases in SRT). For PRO SRT, these occurred from the ages of 23.4–33.1 and 52.2–58.8, and for ANTI SRT, these occurred from the ages of 25.2–29.5, 50.4–57.5, and 74.3–86.5.

#### Pro-saccade Express- and Regular-Latency Correct Responses

**FIGURE 4:**
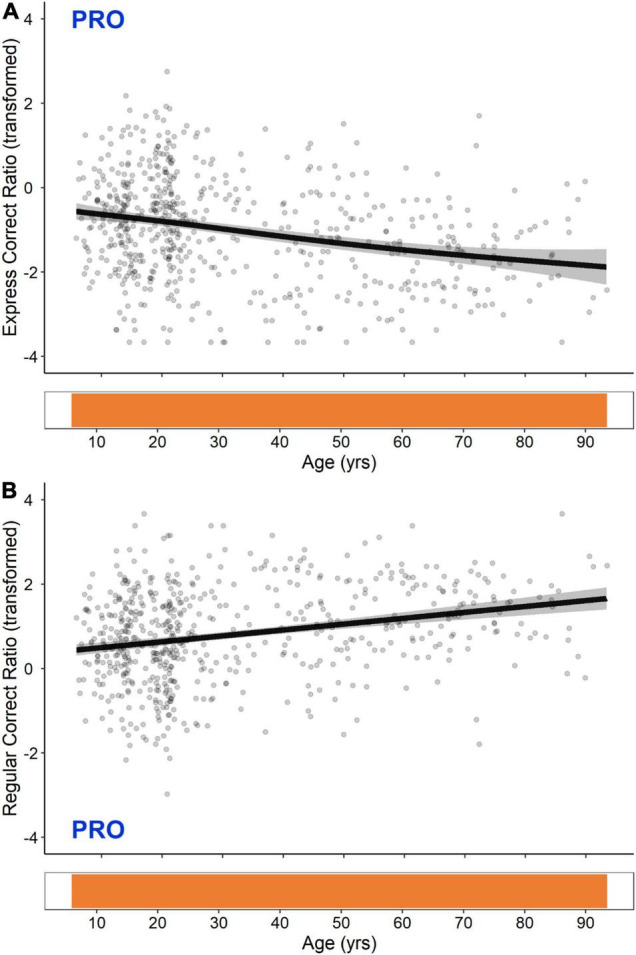
GAM fits and significant periods of change for logit transformed PRO express-latency **(A)** and regular-latency **(B)** correct response ratios. Scatter points are individual participants, black curves are the GAM fits, and gray ribbons are the 95% confidence intervals. Bottom tiles indicate significant periods of change. Note that these are continuous across the entire lifespan.

GAM fits and significant periods of change for PRO express- and regular-latency correct response ratios are shown in Figure 4. These two measures exhibited opposing linear trends that were significant across the entire lifespan; PRO express-latency correct responses decreased continuously from the ages of 5–93, whereas PRO regular-latency correct responses increased continuously from the ages of 5–93.

#### Anti-saccade Express- and Regular-Latency Direction Errors

**FIGURE 5:**
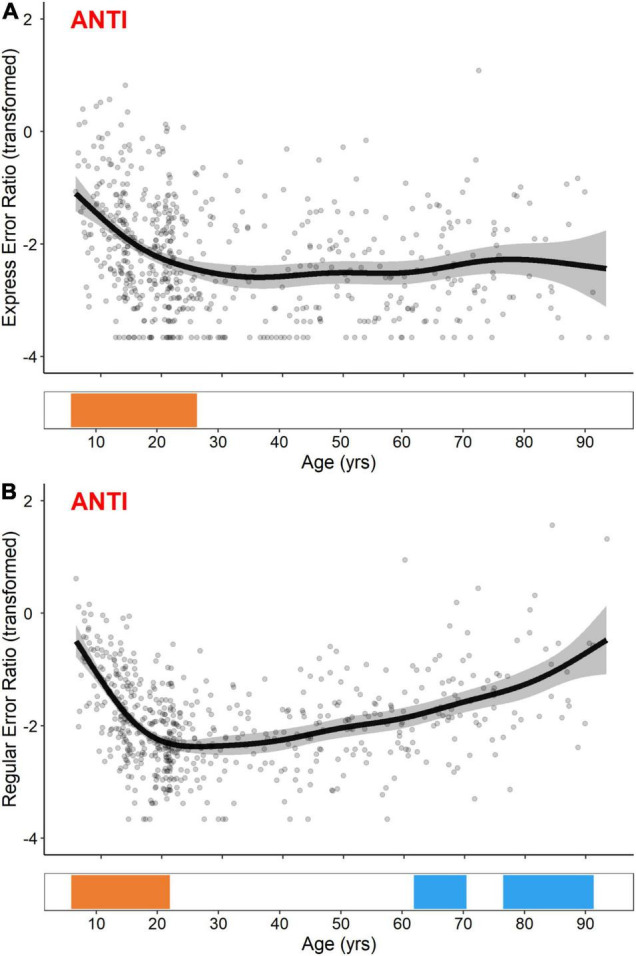
GAM fits and significant periods of change for logit transformed ANTI express-latency **(A)** and regular-latency **(B)** direction error ratios. Scatter points are individual participants, black curves are the GAM fits, and gray ribbons are the 95% confidence intervals. Bottom tiles indicate significant periods of developmental-related (orange) and aging-related (blue) change.

In contrast to PRO express- and regular-latency correct response ratios, GAM fits for ANTI express- and regular-latency direction error ratios were distinctively non-linear (Figure 5). While both measures exhibited significant developmental-related periods of improvement (i.e., decreases in error ratios), occurring from the ages of 5.8–26.4 for ANTI express-latency direction errors and 5.8–22.0 for ANTI regular-latency direction errors, only the ANTI regular-latency direction errors exhibited subsequent aging-related periods of decline (i.e., increases in error ratio) from the ages of 63.7–70.2 and 76.7–89.7.

#### Voluntary Override Time

**FIGURE 6:**
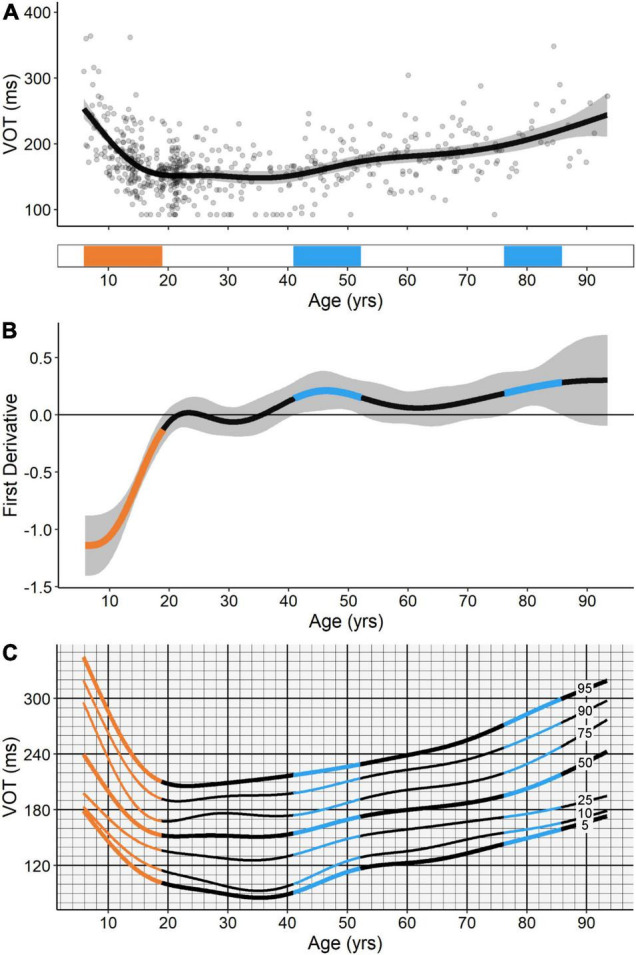
**(A)** GAM fit and significant periods of change for voluntary override time (VOT). Scatter points are individual participants, black curve is the GAM fit, and gray ribbon is the 95% confidence interval. Bottom tile indicates significant periods of developmental-related (orange) and aging-related (blue) change. **(B)** First derivative of the GAM fit shown in panel **(A)**. Negative values indicate improvements in task performance (i.e., decreases in VOT) and positive values indicate declines in task performance (i.e., increases in VOT). Significant periods of developmental-related (orange) and aging-related (blue) change were identified at the ages where the confidence intervals of the first derivative of the GAM did not contain zero (*p* < 0.05). **(C)** A hypothetical “cognitive growth curve” for VOT consisting of the 5th, 10th, 25th, 50th, 75th, 90th, and 95th percentile curves for the measure.

The GAM fit for VOT was also distinctively non-linear (Figure 6A), and, as to be expected, resembled the fitted trends for ANTI viable correct SRT and ANTI regular-latency direction error ratio. VOT exhibited a significant developmental-related period of improvement (i.e., decreases in VOT) from the ages of 5.8–19.0, followed by two significant aging-related periods of decline (i.e., increases in VOT), the first from the ages of 40.9–52.2 and the second from the ages of 76.1–85.6. Relative to the other IPAST measures investigated, age explained the highest proportion of deviance for VOT, at 31.2% (Table 1).

Additional information regarding the rate of change and percentiles for VOT are illustrated in Figures 6B,C, respectively, and also described below. We elaborate upon this measure given that it summarizes both ANTI correct responses and ANTI direction errors for a given individual, and was explained in large part by age in the current study cohort. Figure 6B shows the first derivative of the GAM fit for this measure. Negative values in this plot indicate improvements in task performance (i.e., decreases in VOT), while positive values indicate declines in task performance (i.e., increases in VOT). Although the first derivative of the GAM fit is non-linear across the lifespan, the rate of change can be approximated at -7 ms/year for the initial developmental-related period of improvement, 1 ms/year for the first aging-related period of decline, and 2 ms/year for the second aging-related period of decline. These estimates highlight the sensitivity of VOT to the dynamic improvement, maturation, and decline of inhibitory control across the lifespan. Figure 6C provides the 5th–95th percentile curves for VOT across the lifespan. We propose potential applications for such a “cognitive growth curve” in the Section “Discussion.”

### Relationships Between Interleaved Pro/Anti-saccade Task Measures

**FIGURE 7:**
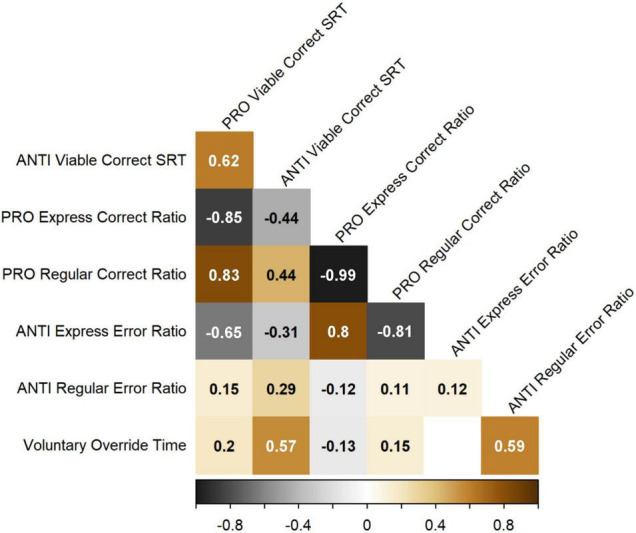
Spearman’s correlation matrix for the seven IPAST measures of interest, after controlling for age. *R* values are shown for the pairwise relationships between each measure, with warm colors indicating positive correlations and cool colors indicating negative correlations. Only significant correlations (*p* < 0.05) are shown.

Standardized residuals derived from each of the GAMs described above were input into Spearman’s correlations to investigate the pairwise relationships between measures after controlling for age (Figure 7). PRO and ANTI viable correct SRT were positively correlated with one another. PRO and ANTI SRT were also both positively correlated with PRO regular-latency correct response ratio and ANTI regular-latency direction error ratio, and negatively correlated with PRO express-latency correct response ratio and ANTI express-latency direction error ratio. PRO express-latency correct response ratio was negatively correlated with PRO regular-latency correct response ratio, and positively correlated with ANTI express-latency direction error ratio. Finally, VOT was positively correlated with both ANTI viable correct SRT and ANTI regular-latency direction error ratio, as expected. All reported correlations were statistically significant (*p* < 0.05).

## Discussion

This is the first study to use IPAST data collected from a large cross-sectional cohort of normative individuals, standardized pre-processing protocols, and flexible modeling techniques to robustly characterize changes in inhibitory control across the lifespan. As hypothesized, GAM fits for the majority of the measures investigated followed a curvilinear U-shaped trajectory that has previously been reported in studies assessing cognitive performance (Cepeda et al., 2001; Bedard et al., 2002; Ferguson et al., 2021), and its relationship to underlying brain structure and function (Danielsen et al., 2020; Kupis et al., 2021) across the lifespan. Change point analysis provided further insight into significant periods of change occurring along these trajectories. While PRO express- and regular-latency correct responses exhibited continuous linear change across the entire lifespan, PRO and ANTI viable correct SRT, ANTI express- and regular-latency direction errors, and VOT all matured throughout adolescence. Subsequent decline began in the mid-20s for PRO and ANTI SRT, in the 40s for VOT, and in the 60s for ANTI regular-latency direction errors. We discuss these dynamic behavioral changes in relation to converging animal, human, and computational literature which provides insight into underlying neural mechanisms.

### Pro-saccade and Anti-saccade Viable Correct Saccadic Reaction Time

GAM fits of PRO and ANTI viable correct SRT demonstrated that PRO SRT decreased from the ages of 5–17 and increased from the ages of 23–33 and 52–58, while ANTI SRT decreased from the ages of 5–18 and increased from the ages of 25–29, 50–57, and 74–86 (Figure 3). The initial dramatic decrease in PRO and ANTI SRT captured here is consistent with past group- and regression-based studies, reporting that saccade latencies decrease significantly from the ages of 5–25 (Munoz et al., 1998; Fukushima et al., 2000; Klein and Foerster, 2001; Luna et al., 2004; Kramer et al., 2005; Velanova et al., 2008; Ordaz et al., 2010; Bucci and Seassau, 2012; Alahyane et al., 2014). It has previously been proposed that PRO and ANTI SRT reach adult-levels by the ages of 12–14 (Fukushima et al., 2000; Klein and Foerster, 2001; Irving et al., 2006; Bucci and Seassau, 2012). Using a large cohort of 8–30 year olds, blocked PRO/ANTI paradigm, and change point analysis based on fitted piecewise linear models, Luna et al. (2004) identified 15 as the age at which saccade latencies reached adult-levels. Our findings of saccade latencies maturing later in adolescence (i.e., 17 and 18) likely reflects our use of: (1) a larger study cohort spanning the entire lifespan, (2) a more cognitively demanding eye tracking paradigm (i.e., interleaved vs. blocked design), and (3) more flexible analytical approaches, relative to past work.

Our findings of gradual increases in PRO and ANTI SRT during aging is consistent with numerous group-based studies describing significant differences in SRT between adults in the third and fourth decades of life relative to those in the seventh, eighth, and ninth (Munoz et al., 1998; Klein et al., 2000; Sweeney et al., 2001; Abel and Douglas, 2007; Fujiwara et al., 2010; Bonnet et al., 2013; Noiret et al., 2016). Two recent, well-powered regression-based studies further determined that PRO and ANTI SRT increased significantly from the ages of 30–95 (Coors et al., 2021) and 51–84 (Mack et al., 2020). Our change point analysis revealed that PRO and ANTI SRT begin to increase as early as the ages of 23 and 25, respectively. While there is a paucity of studies examining PRO and ANTI behavior in adults from the third through fifth decades of life, studies investigating the effect of age on other behavioral paradigms of inhibitory control and processing speed have similarly suggested that decline may begin as early as the 20–30s (Salthouse, 2009; Ferguson et al., 2021).

As previously introduced, a considerable advantage to using the IPAST to investigate inhibitory control across the lifespan is that behaviors can be linked to well-characterized frontal-parietal and frontal-striatal mechanisms. Monkey neurophysiology, human lesion, and human neuroimaging work have jointly indicated that the frontal, supplementary, and parietal eye fields (FEF, SEF, PEF) are critical in the planning, sensorimotor mapping, and execution of saccades (Connolly et al., 2002; Pierrot-Deseilligny et al., 2002; Amador et al., 2004; Munoz and Everling, 2004). Notably, preparatory FEF activity prior to STIM appearance on PRO and ANTI trials predicts subsequent saccade latencies in both monkeys (Everling and Munoz, 2000) and humans (Connolly et al., 2005; Alahyane et al., 2014; Fernandez-Ruiz et al., 2018). Previous studies employing event-related fMRI during IPAST performance have shown that children have significantly lower preparatory FEF, SEF, and PEF activity relative to adolescents and adults (Alahyane et al., 2014), while older adults have significantly lower SEF activity relative to younger adults (Fernandez-Ruiz et al., 2018). Our findings of PRO and ANTI SRT maturation at the ages of 17 and 18, as well as their subsequent decline beginning in the mid-20s may therefore be attributed to changes in the structural and functional integrity of the FEF, SEF, and PEF.

### Pro-saccade and Anti-saccade Express- and Regular-Latency Behavior

PRO express- and regular-latency correct responses exhibited opposing linear trends that were significant across the entire lifespan (Figure 4). Using a blocked PRO/ANTI paradigm, we have previously reported higher express-latency saccades in individuals younger than 40 (Munoz et al., 1998), and others have found express-latency saccade rates to be significantly higher in 6–7 and 10–11 year olds relative to 18–26 year olds (Klein and Foerster, 2001), and in 20–35 year olds relative to 59–73 and 74–88 year olds (Klein et al., 2000). Most comparable to the present results, Klein et al. (2005) also found a weak negative linear relationship between age and express-latency saccade rate in a cohort of individuals aged 9–88.

PRO express- and regular-latency correct responses are both triggered by the STIM visual transient. Express-latency responses result from a direct sensory-motor transformation of the transient (Edelman and Keller, 1996; Dorris et al., 1997; Sparks et al., 2000) that occurs in the SC (Schiller et al., 1987), while regular-latency responses result from the propagation of a well-learned, automated motor command that may involve cortical areas such as the PEF (Munoz and Everling, 2004; Coe and Munoz, 2017). Our findings suggest that with age, cortically mediated automated processes may increasingly dominate subcortically mediated reflexive processes, resulting in a continuous decrease in the proportion of express- to regular-latency responses.

ANTI express- and regular-latency direction errors exhibited distinctly non-linear trends across the lifespan; both error types decreased until the mid-20s, but only regular-latency errors subsequently increased beginning at the age of 63 (Figure 5). It is well-established that children make more direction errors than adolescents, who make more direction errors than young adults (Fukushima et al., 2000; Klein and Foerster, 2001; Luna et al., 2004; Kramer et al., 2005; Velanova et al., 2008; Ordaz et al., 2010; Bucci and Seassau, 2012; Alahyane et al., 2014), and that older adults make more direction errors than younger adults (Klein et al., 2000; Sweeney et al., 2001; Abel and Douglas, 2007; Fujiwara et al., 2010; Peltsch et al., 2011; Bonnet et al., 2013; Noiret et al., 2016; Fernandez-Ruiz et al., 2018; Mack et al., 2020; Coors et al., 2021). The latency of these direction errors, however, is rarely characterized. In one of the few studies to do so, Klein and Foerster (2001) found that the difference in the proportion of express- to regular-latency direction errors was significantly greater in 6–7 year olds relative to 10–11 and 18–26 year olds. While not explicitly examining express- and regular-latencies, other studies have described longer direction error latencies in older relative to younger adults (Bowling et al., 2012; Noiret et al., 2016).

On ANTI trials, pre-emptive, global inhibition provided by regions such as the DLPFC, FEF, SEF, BG, and SC is required prior to STIM appearance to suppress the express-latency direction error (Coe and Munoz, 2017; Coe et al., 2019). Subsequently, coordinated activity between these regions is required to drive the voluntary, location-specific motor command for an ANTI to overcome the regular-latency direction error. Support for these claims stems from monkey (Everling et al., 1999; Everling and Munoz, 2000; Amador et al., 2004; Johnston and Everling, 2006; Johnston et al., 2007; Watanabe and Munoz, 2010) and human (Connolly et al., 2002; Curtis and D’Esposito, 2003; DeSouza et al., 2003; Ford et al., 2005; Brown et al., 2007) work demonstrating differential preparatory activity in these regions on ANTI vs. PRO trials. Preparatory activity in the DLPFC and anterior cingulate cortex (ACC) has been specifically associated with the monitoring and suppression of direction errors on ANTI trials (Pierrot-Deseilligny et al., 2002, 2003; Ford et al., 2005; Johnston and Everling, 2006; Johnston et al., 2007).

Neuroimaging studies of PRO and ANTI behavior in development suggest that functional activity and effective connectivity of frontal-parietal and frontal-striatal regions become more widely distributed from childhood through to adulthood, supporting a reduction in direction error rates (Luna et al., 2001; Velanova et al., 2008; Hwang et al., 2010; Ordaz et al., 2013; Alahyane et al., 2014). Although neuroimaging studies of PRO and ANTI behavior in aging cohorts have reported mixed findings to-date (Raemaekers et al., 2006; Nelles et al., 2009; Alichniewicz et al., 2013), Fernandez-Ruiz et al. (2018) recently found that older adults had significantly lower preparatory SEF and ACC activity relative to younger adults, potentially contributing to a reduced ability to monitor task performance and drive the voluntary motor command for a correct ANTI. Considering the present work, the ability to suppress both express- and regular-latency direction errors by the mid-20s may be mediated by mature activation and integration of frontal-parietal and frontal-striatal regions, including the DLPFC, FEF, SEF, BG, and SC. By the 60s, however, the ability of these regions to drive the voluntary motor command for a correct ANTI may begin to deteriorate, resulting in an increase in regular-latency direction errors.

### Voluntary Override Time

We characterized the time at which voluntary processes begin to overcome automatic processes on ANTI trials, or the VOT (Coe et al., 2019), for the first time across the lifespan. VOT decreased dramatically from the ages of 5–19, remained relatively stable through the third and fourth decades of life, then exhibited gradual increases from the ages of 40–52 and 76–85 (Figure 6A). Recent work from our group describes a generative model of saccadic action selection, inspired by known signal components of neural activity, capable of producing PRO and ANTI behaviors similar to those observed here (Coe et al., 2019). *Post-hoc* investigations into the VOT generated by this model revealed that simulating a type of inhibitory “crosstalk” between voluntary (based on activity in the FEF, SEF, and SC) and automated (based on activity in the PEF) signals, such that voluntary signals could override automated signals, produced behavior that better approximated human data relative to prior simulations (Coe et al., 2019). The present findings indicate that the ability of voluntary signals to override automated signals matures at the age of 19 (i.e., after maturation of saccade latency but before maturation of direction error suppression), and begins to decline at the age of 40 (i.e., after decline of saccade latency but before decline of direction error suppression). Taken together, the animal, human, and computational literature described here provide insight into potential frontal-parietal and frontal-striatal mechanisms underlying the behavioral changes revealed in our analysis. We suggest that structural and functional maturation of these circuits mediates decreased saccade latencies and direction error rates throughout adolescence, while their subsequent decline mediates increased saccade latencies in the mid-20s and increased regular-latency errors in the 60s. Increasing input from cortical, relative to subcortical regions of the brain mediates a continuous decrease in the proportion of PRO express- to regular-latency correct responses across age.

### Limitations and Future Directions

The generalizability of our study cohort is somewhat limited by the overrepresentation of young female participants. Recent work has highlighted the importance of having sufficient numbers of middle-aged adults in lifespan cohorts in order to more comprehensively characterize cognitive changes that occur with age (Ferguson et al., 2021). As there is a paucity of studies investigating PRO and ANTI behavior in adults from the third through fifth decades of life, our findings of PRO and ANTI SRT beginning to decline as early as the mid-20s requires replication. The uneven distribution of female and male participants in our study cohort prevented us from conducting an in-depth analysis of sex differences in IPAST behavior. However, BIC values indicated that GAMs specified with a smoothed fixed effect of age provided a superior fit for all measures relative to GAMs specified with a smoothed fixed effect of age split by sex. Previous studies examining sex differences in PRO and ANTI tasks have been mixed; some failing to identify any differences (Sweeney et al., 2001; Fujiwara et al., 2010; Bonnet et al., 2013), others reporting shorter latencies in adolescent females relative to males (Luna et al., 2004), and others reporting longer latencies in adult females relative to males (Bargary et al., 2017; Mack et al., 2020). Although sex differences in PRO and ANTI behavior remains to be clarified, it is clear that any sex effects that do exist are substantially weaker relative to those of age (Ordaz et al., 2018; Coors et al., 2021).

A second limitation of the present work is the reliance on a cross-sectional study design rather than a longitudinal one, as the latter is more sensitive to age-related changes and inter-individual variability (Casey et al., 2005; Goh et al., 2012; Sørensen et al., 2021). Indeed, the GAM and change point analyses described here are highly amenable to longitudinal study designs (Simmonds et al., 2014; Wierenga et al., 2019; Calabro et al., 2020; Danielsen et al., 2020). Regarding inter-individual variability, there are likely many biological, environmental, and psychosocial factors contributing to variability in IPAST behavior that have yet to be fully characterized (Bargary et al., 2017). As the frequency and timing of express-latency saccades can be influenced by various task parameters (Dorris and Munoz, 1998; Bibi and Edelman, 2009; Marino and Munoz, 2009), it is likely that these behaviors also vary on an individual participant basis. Investigation into the factors mediating inter-individual variability of the frequency and timing of express-latency saccades is therefore a promising direction of future work.

The GAM fits and significant periods of change presented here have significant implications for improving our understanding of the vulnerable windows of brain maturation and decline in which the risk for developing neurological disorders may be elevated. Attention-deficit hyperactivity disorder and Parkinson’s disease are two neurological disorders with onset at either ends of the lifespan that can be differentiated from healthy age-matched controls based on ANTI SRT and ANTI express- and regular-latency direction errors (Cameron et al., 2012; Hakvoort Schwerdtfeger et al., 2013; Coe and Munoz, 2017). As VOT indexes both of these ANTI behaviors and is explained in large part (31%) by age, this measure presents as an ideal “cognitive growth curve” for differentiating normative aging and neurological disease across the lifespan (Figure 6C). We propose that this curve could be used to identify individuals in early childhood and late adulthood who may be at an elevated risk for developing these disorders, by virtue of their VOT values falling outside of a specified percentile for normative aging.

## Conclusion

The characterization of inhibitory control across the lifespan is critical if we hope to improve our understanding of the vulnerable windows of brain maturation and decline in which the risk for developing neurological disorders is elevated. The GAM fits presented here expand upon previous eye tracking literature by capturing complex age-related processes within continuous, flexible models that also enabled us to identify the ages at which significant change occurred. Drawing on converging animal, human, and computational literature, we propose potential frontal-parietal and frontal-striatal mechanisms that may mediate the behavioral changes revealed in our analysis. Future work which focuses on longitudinal assessment and investigation into the inter-individual factors contributing to inhibitory control will be paramount in furthering our understanding of the neural mechanisms that differ in healthy aging and neurological disease.

## Data Availability Statement

The raw data supporting the conclusions of this article will be made available by the authors, without undue reservation.

## Ethics Statement

The studies involving human participants were reviewed and approved by Queen’s University Health Sciences and Affiliated Teaching Hospitals Research Ethics Board. Written informed consent to participate in this study was provided by the participants’ legal guardian/next of kin.

## Author Contributions

DM, BC, DB, and MS conceived the study. RY, MS, HR, OC, RK, and JP contributed to data collection. RY and HR organized and verified the dataset. BC and DB wrote code that was implemented in various stages of data archiving, pre-processing, and analysis. DB wrote the IPAST experimental code and set up the hardware for data collection. BC created the standardized pipeline used for all IPAST pre-processing and analysis. RY conducted the generalized additive modeling and change point analysis. RY and DM wrote the manuscript. JH, BC, DB, and DM provided support and feedback throughout the data analysis and manuscript preparation process. All authors contributed to the article and approved the submitted version.

## Conflict of Interest

The authors declare that the research was conducted in the absence of any commercial or financial relationships that could be construed as a potential conflict of interest.

## Publisher’s Note

All claims expressed in this article are solely those of the authors and do not necessarily represent those of their affiliated organizations, or those of the publisher, the editors and the reviewers. Any product that may be evaluated in this article, or claim that may be made by its manufacturer, is not guaranteed or endorsed by the publisher.

## Abbreviations

ACC: anterior cingulate cortex
ANTI: anti-saccade
BG: basal ganglia
BIC: Bayesian information criterion
DLPFC: dorsolateral prefrontal cortex
FEF: frontal eye fields
FP: fixation point
GAM: generalized additive model
IPAST: interleaved pro/anti-saccade task
ITI: inter-trial interval
MoCA: Montreal Cognitive Assessment
PEF: parietal eye fields
PRO: pro-saccade
REML: restricted marginal likelihood maximization
SC: superior colliculus
SEF: supplementary eye fields
SRT: saccadic reaction time
STIM: stimulus
VOT: voluntary override time.
