# Supplementary figures and images for: Interleaved Pro/Anti-saccade Behavior Across the Lifespan
Source: Front Aging Neurosci. 2022 May 18;14:842549. doi: 10.3389/fnagi.2022.842549 (PMC9159803; doi:10.3389/fnagi.2022.842549)

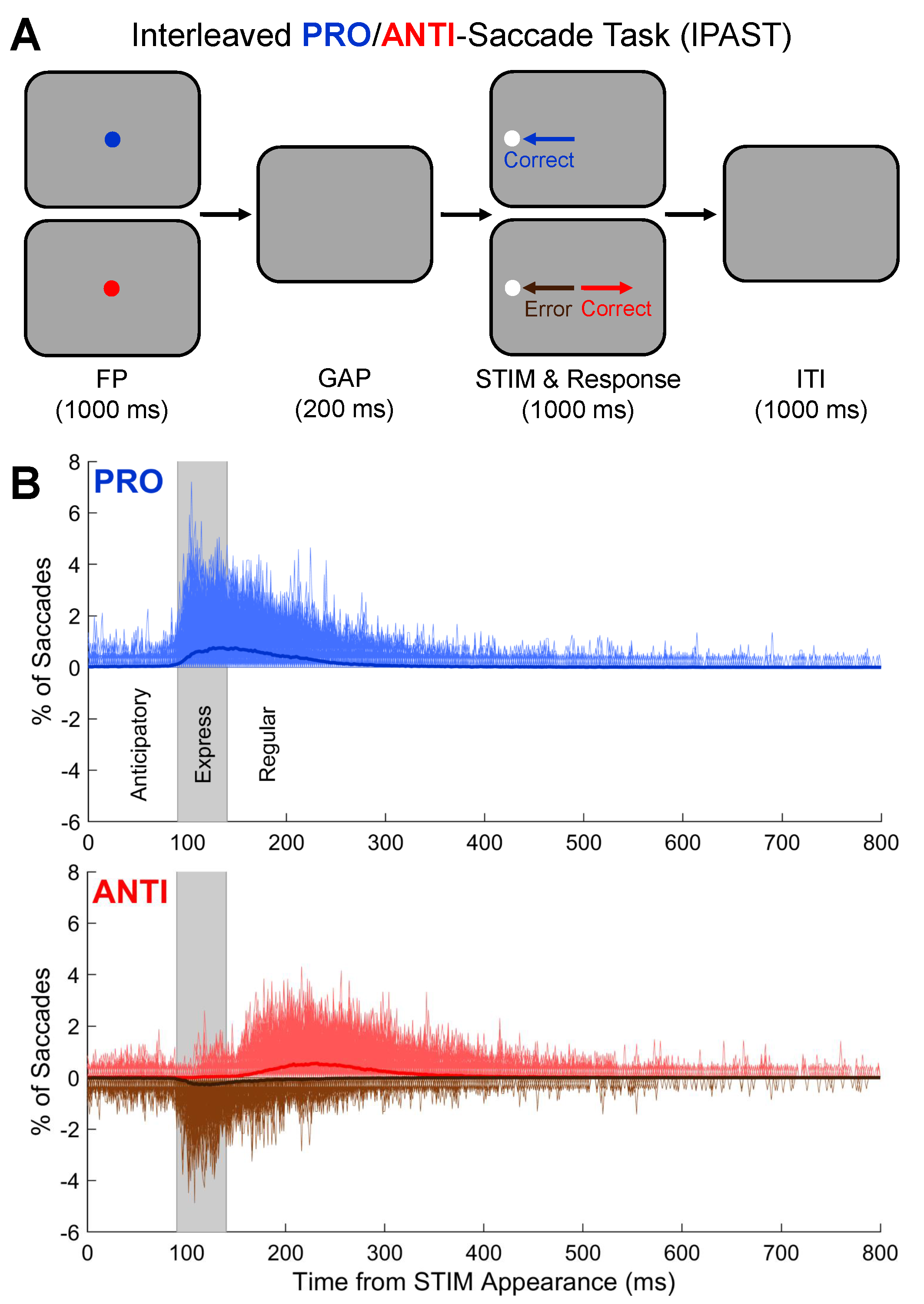

Supplement: Supplementary file 1 [file Image_1.TIFF]

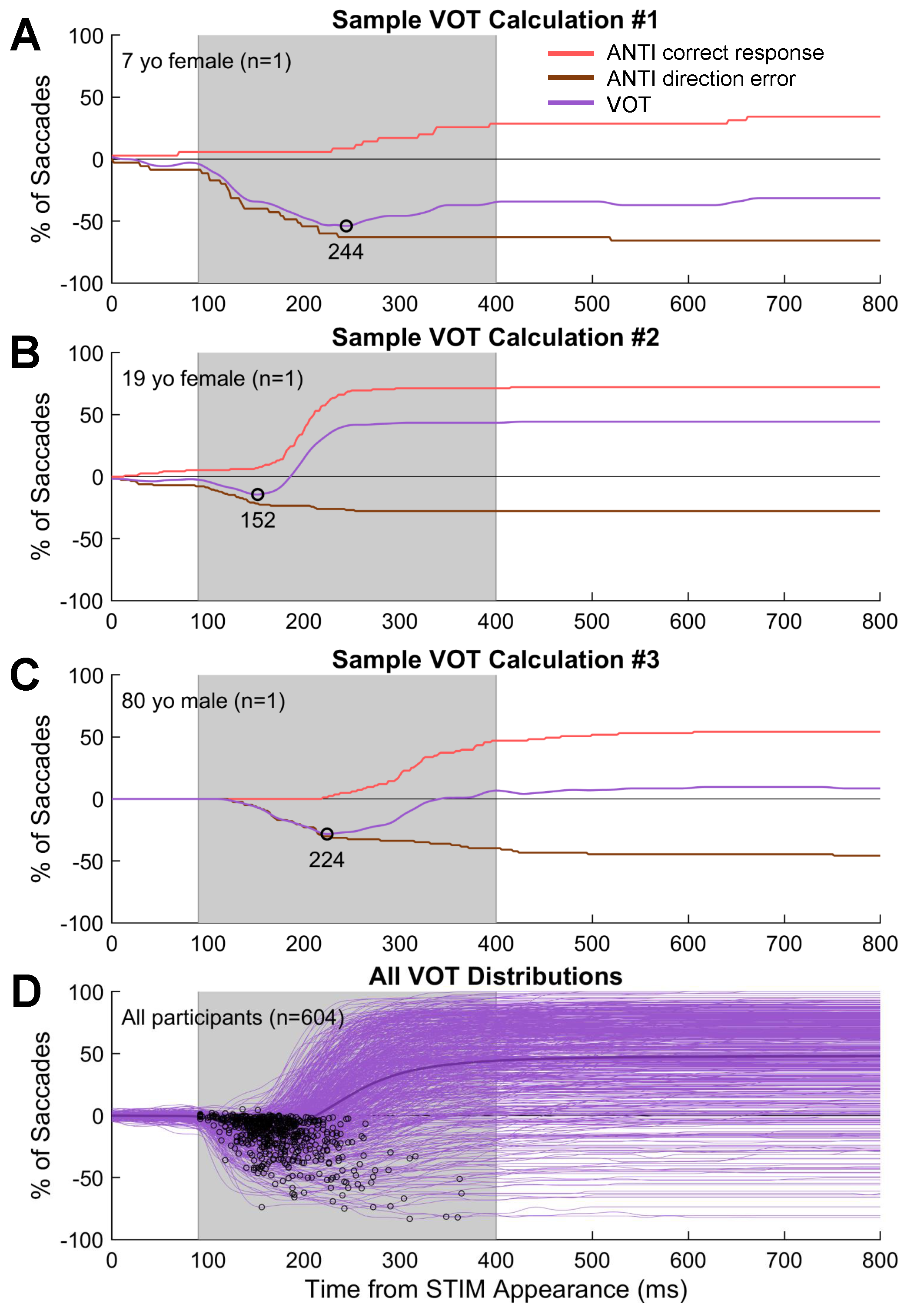

Supplement: Supplementary file 2 [file Image_2.TIFF]
