# Supplementary material for: Interleaved Pro/Anti-saccade Behavior Across the Lifespan
Source: Front Aging Neurosci. 2022 May 18;14:842549. doi: 10.3389/fnagi.2022.842549 (PMC9159803; doi:10.3389/fnagi.2022.842549)
Supplement: Supplementary file 3 [file Table_1.DOCX]

**Supplementary Table 1**

Demographic information for included (INC) and excluded (EXC) study participants. Age bins are as in Fig. 2A. ORS = outlier rejection step. * indicates MoCA scores that resulted in ORS3 exclusion.

| Age Bin | Study Status (n) | Sex (n) | Education (n) | Mean/Individual MoCA Score  (n) | Reason for Exclusion (n) |
| --- | --- | --- | --- | --- | --- |
| 5-8 | INC (22) | F (11); M (11) | Elementary (22) | N/A | N/A |
|  | EXC (5) | F (3); M (2) | Elementary (5) | N/A | ORS1 (3); ORS2 (2) |
| 9-11 | INC (38) | F (16); M (22) | Elementary (38) | N/A | N/A |
|  | EXC (4) | F (1); M (3) | Elementary (4) | N/A | ORS1 (1); ORS2 (2);  Other (1) |
| 12-14 | INC (84) | F (50); M (34) | Elementary (84) | N/A | N/A |
|  | EXC (3) | F (2), M (1) | Elementary (3) | N/A | ORS2 (3) |
| 15-17 | INC (41) | F (29); M (12) | High School (41) | N/A | N/A |
|  | EXC (4) | F (3); M (1) | High School (4) | N/A | ORS2 (4) |
| 18-20 | INC (77) | F (54); M (23) | High School (24);  College (2);  Undergraduate (51) | 28.02 (52) | N/A |
| 21-25 | INC (96) | F (66); M (30) | High School (7);  College (3);  Undergraduate (57);  Graduate (22);  Professional (2) | 28.03 (58) | N/A |
| 26-30 | INC (31) | F (19); M (12) | High School (1);  College (3);  Undergraduate (8);  Graduate (17);  Professional (2) | 28.33 (27) | N/A |
| 31-35 | INC (17) | F (13); M (4) | High School (2);  College (5);  Undergraduate (4);  Graduate (6) | 28.06 (15) | N/A |
| 36-40 | INC (18) | F (16); M (2) | College (5);  Undergraduate (5);  Graduate (7);  Professional (1) | 28.73 (15) | N/A |
| 41-45 | INC (25) | F (19); M (6) | High School (2);  College (5);  Undergraduate (9);  Graduate (8);  Professional (1) | 28.13 (24) | N/A |
| 46-50 | INC (27) | F (20); M (7) | High School (1);  College (8);  Undergraduate (10);  Graduate (7);  Professional (1) | 28.62 (26) | N/A |
| 51-55 | INC (19) | F (8); M (11) | High School (3);  College (7);  Undergraduate (4);  Graduate (4);  Professional (1) | 28.21 (19) | N/A |
|  | EXC (2) | F (1); M (1) | College (1); Graduate (1) | 26 (1); 26 (1) | ORS2 (2) |
| 56-60 | INC (23) | F (14); M (9) | High School (3);  College (9);  Undergraduate (6);  Graduate (3);  Professional (2) | 27.91 (23) | N/A |
| 61-65 | INC (19) | F (16); M (3) | High School (1);  College (10);  Undergraduate (7);  Professional (1) | 28 (16) | N/A |
| 66-70 | INC (24) | F (17); M (7) | High School (3);  College (7);  Undergraduate (6);  Graduate (6);  Professional (2) | 27.25 (20) | N/A |
| 71-75 | INC (18) | F (12); M (6) | High School (3);  College (4);  Undergraduate (5);  Graduate (4);  Professional (2) | 27.13 (15) | N/A |
|  | EXC (2) | F (1); M (1) | High School (1); Graduate (1) | *18 (1); 22 (1) | ORS1 (1); ORS3 (1) |
| 76-80 | INC (9) | F (4); M (5) | High School (1);  College (3);  Undergraduate (2);  Graduate (2);  Professional (1) | 27.22 (9) | N/A |
| 81-85 | INC (7) | F (4); M (3) | High School (2);  Undergraduate (4);  Professional (1) | 25.71 (7) | N/A |
|  | EXC (2) | F (1); M (1) | Undergraduate (1); Graduate (1) | 23 (1); 25 (1) | ORS1 (1); ORS2 (1) |
| 86-90 | INC (7) | F (3); M (4) | High School (1);  Undergraduate (6); | 25.14 (7) | N/A |
|  | EXC (4) | F (3); M (1) | Elementary (1);  Undergraduate (3) | *16 (1); *19 (1); 26 (1); 29 (1) | ORS1 (2); ORS3 (2) |
| 91-93 | INC (2) | F (2) | High School (1);  Graduate (1); | 21 (1); 26 (1) | N/A |
|  | EXC (1) | F (1) | Undergraduate (2) | 26 (1) | ORS1 (1) |
