# Supplementary material for: Interleaved Pro/Anti-saccade Behavior Across the Lifespan
Source: Front Aging Neurosci. 2022 May 18;14:842549. doi: 10.3389/fnagi.2022.842549 (PMC9159803; doi:10.3389/fnagi.2022.842549)
Supplement: Supplementary file 4 [file Table_2.DOCX]

**Supplementary Table 2**

GAM fit parameters. Lower BIC values (shown in bold) indicate the model with superior fit.

| IPAST Measure | Model | *Ref df* | *F* | *p* | *R^2^* | Deviance Explained | BIC |
| --- | --- | --- | --- | --- | --- | --- | --- |
| PRO  viable correct SRT | 1 | 6.925 | 10.74 | <2e-16 | 0.11 | 11.8% | **6128.941** |
|  | 2  (F, M) | 2.306,  5.372 | 17.416,  5.482 | < 2e-16,  5.25e-05 | 0.101 | 11.2% | 6146.209 |
| ANTI  viable correct SRT | 1 | 8.412 | 25.22 | <2e-16 | 0.26 | 26.9% | **6332.529** |
|  | 2  (F, M) | 7.945,  7.238 | 13.37,  13.66 | <2e-16,  <2e-16 | 0.254 | 27.1% | 6377.061 |
| PRO  express-latency correct response ratio | 1 | 1.998 | 26.04 | <2e-16 | 0.079 | 8.15% | **1938.788** |
|  | 2  (F, M) | 1.003,  1.880 | 36.785,  7.693 | < 2e-16,  0.000579 | 0.0767 | 8.21% | 1950.467 |
| PRO  regular-latency correct response ratio | 1 | 1.005 | 46.33 | <2e-16 | 0.0703 | 7.18% | **1816.082** |
|  | 2  (F, M) | 1.001,  1.004 | 33.74,  12.90 | < 2e-16,  0.000349 | 0.0685 | 7.31% | 1828.029 |
| ANTI  express-latency direction error ratio | 1 | 6.106 | 13.9 | <2e-16 | 0.124 | 13.1% | **1659.440** |
|  | 2  (F, M) | 5.217,  4.421 | 8.864,  8.291 | < 2e-16,  1.4e-06 | 0.118 | 13.1% | 1686.447 |
| ANTI  regular-latency direction error ratio | 1 | 7.857 | 31.88 | <2e-16 | 0.293 | 30.1% | **1317.410** |
|  | 2  (F, M) | 7.491,  6.493 | 18.30,  17.03 | <2e-16,  <2e-16 | 0.29 | 30.5% | 1355.761 |
| Voluntary override time | 1 | 8.429 | 31.23 | <2e-16 | 0.303 | 31.2% | **6064.501** |
|  | 2  (F, M) | 8.193,  6.921 | 17.43,  16.69 | <2e-16,  <2e-16 | 0.299 | 31.5% | 6106.566 |
