# Supplementary material for: Interleaved Pro/Anti-saccade Behavior Across the Lifespan
Source: Front Aging Neurosci. 2022 May 18;14:842549. doi: 10.3389/fnagi.2022.842549 (PMC9159803; doi:10.3389/fnagi.2022.842549)
Supplement: Supplementary file 5 [file Data_Sheet_1.DOCX]

Supplementary Material

**SUPPLEMENTARY FIGURE CAPTIONS**

**Supplementary Figure 1. A.** Visual representation of the Interleaved PRO/ANTI-Saccade Task (IPAST). Each trial began with the appearance of a fixation point (FP) in the center of a black screen for 1000 ms. The color of the FP indicated the trial condition (green=PRO, red=ANTI). Following a 200 ms gap (GAP) during which the FP was removed, a gray stimulus (STIM) appeared 10° to the left or right of the FP position and remained on screen for an additional 1000 ms. On PRO trials, participants were instructed to look at the STIM as soon as it appeared. On ANTI trials, participants were instructed to look away from the STIM as soon as it appeared. Direction errors were saccades made toward the STIM on ANTI trials. An inter-trial interval (ITI) was presented for 1000 ms before the start of each new trial. Note that for illustration purposes, the colors of the FP, screen, and STIM shown in **A** differ slightly from how the task would appear to participants in the lab. **B.** Instantaneous SRT distributions for PRO and ANTI trials. On PRO and ANTI trials, saccades were classified based on when they occurred and their start and end positions. Saccades made towards the two potential STIM locations occurring between -110–89 ms relative to STIM appearance were considered “anticipatory” and excluded from further analysis. Saccades made towards the two potential STIM locations occurring between 90­–800 ms relative to STIM appearance were considered “viable” and further delineated based on their latencies. PRO viable correct responses and ANTI viable direction errors were divided into express (90–139 ms) and regular (140–800 ms) latencies. Thick lines are averaged distributions for the entire study cohort. Thin lines are individual participants. Vertical gray windows indicate the express-latency epoch.

**Supplementary Figure 2. A-C.** Sample voluntary override time (VOT) calculations for three exemplar participants spanning childhood **(A),** adolescence **(B),** and old age **(C).** Cumulative VOT distributions (purple curves) were calculated by subtracting the cumulative SRT distributions of ANTI direction errors (brown curves) from the cumulative SRT distributions of ANTI correct responses (red curves). Resulting distributions were smoothed with a 7-point box shaped kernel. VOT for each participant was determined as the minimum point along these smoothed distributions (black circles) occurring within 90–400 ms (vertical gray window) relative to STIM appearance. **D.** Smoothed cumulative VOT distributions for all participants. Thick line is the averaged distribution for the entire study cohort. Thin lines are individual participants. Vertical gray window indicates the VOT epoch.
